# Effect of resveratrol and combination of resveratrol and donepezil on the expression of microglial cells and astrocytes in *Wistar* albino rats of colchicine-induced Alzheimer’s disease

**DOI:** 10.1007/s13205-023-03743-4

**Published:** 2023-08-26

**Authors:** Y. Lakshmisha Rao, B. Ganaraja, Pooja K. Suresh, Teresa Joy, Sheetal D. Ullal, Poornima A. Manjrekar, B. V. Murlimanju, B. Gaurav Sharma

**Affiliations:** 1 Department of Anatomy, Kasturba Medical College, Mangalore, Manipal Academy of Higher Education, Manipal, Karnataka India; 2 Department of Physiology, Kasturba Medical College, Mangalore, Manipal Academy of Higher Education, Manipal, Karnataka India; 3 Department of Pathology, Kasturba Medical College, Mangalore, Manipal Academy of Higher Education, Manipal, Karnataka India; 4 Department of Anatomy, American University of Antigua College of Medicine, University Park, Jabberwock Beach Road, Coolidge, Antigua, West Indies Antigua and Barbuda; 5 Department of Pharmacology, Kasturba Medical College, Mangalore, Manipal Academy of Higher Education, Manipal, Karnataka India; 6 Department of Biochemistry, Kasturba Medical College, Mangalore, Manipal Academy of Higher Education, Manipal, Karnataka India; 7 Senior Registrar in Trauma and Orthopaedic Surgery, Hampshire Hospitals NHS Foundation Trust, Basingstoke and North Hampshire Hospital, Aldermaston Road, Basingstoke, RG24 9NA UK

**Keywords:** Astrocytes, Healthy brain, Microglia, Neurodegeneration

## Abstract

**Aim:**

The goal was to evaluate the effect of resveratrol (RS) and combination therapy of RS and donepezil (DPZ), on the numerical expression of microglial cells and astrocytes, in the frontal cortex, regions of the hippocampus in colchicine-induced Alzheimer’s disease (AD) model.

**Methods:**

The study involved male albino *Wistar* rats of three months, age and consisted of 6 groups, with six animals each. The immunohistochemical staining with mouse monoclonal anti-human CD 68 and mouse monoclonal anti-GFAP was performed to assess the number of microglial cells and astrocytes, respectively.

**Results:**

AD group showed an increase in the number of microglia, and the numbers declined in the treatment groups, RS 10, RS 20, RS10/10 and DPZ + RS (*p* < 0.001). Astrocyte count was increased in the treatment groups in contrast to the AD group (*p* < 0.05). The DPZ + RS combination group revealed substantial elevation in the number of astrocytes and decreased microglial number among all the groups (*p* < 0.001).

**Conclusion:**

RS administration has diminished the microglial number and elevated the number of astrocytes. The elevated reactive astrocytes have decreased the microglial population. However, the limitation of our study is utilizing the colchicine for the induction of neurodegeneration. Using the transgenic models of AD may give a better insight into the pathogenesis and effect of RS. Another limitation of this study is the administration of RS and DPZ through different routes. The prospects of this research include studying the probiotic nature of RS and the effect of RS in other neurodegenerative disorders.

## Introduction

Alzheimer’s disease (AD) is an advanced neuronal disorder, involving neurodegeneration and death of neurons. Antioxidant properties of resveratrol (RS) have been well known; however, the function of RS in prevention and the advancement of AD are not extensively studied. Particularly, the dose-dependent effect of RS is not much reported previously. Donepezil (DPZ) is an American Food and Drug Administration (FDA) permitted drug used to manage dementia in AD. It is an acetyl-cholinesterase (AChE) inhibitor having a nucleus of benzyl piperidine variety, which is directed toward the catalytic active site (Tripathi et al. 2019). Since the etiology of AD is multifactorial, the multitargeted therapeutic strategy may be more beneficial. The combination of RS and DPZ in the treatment of AD has not being adequately studied and reported. So the current research intends to estimate the role of RS and RS and DPZ combination therapy in the prevention and therapeutic measure in the AD.

A thorough search of the literature yielded little information on neuroglial functions and population in the pathogenesis of AD. A potent antioxidant RS can improve cognitive functions. However, studies are not done for its role in preventing AD by acting on the neuroglial cells. Astrocytes and microglia are essentially required to maintain neural circuits and synaptic homodynamics. The astrocytic reaction in various types of dementia and AD involves both astrogliosis and apoptosis (dystrophy). Astrocytes support synaptogenesis by helping sprouting of axonal and dendritic spines (Arranz and De Strooper 2019; Hampel et al. 2020). Microglia are essential for sensing the pathological events in the brain (Harry 2013). Astrocytes provide local support to the neurons and maintain the formation of synapses. They also help in brain functioning by their phagocytic action (Chung et al. 2013; Liddelow et al. 2017). However, the exact participation of astrocytes and microglia in neuronal degeneration needs to be clearly understood (Anderson et al. 2016; Sanchez-Mejias et al. 2016).

Microglia release the pro-inflammatory mediators like interleukin-β, NO, TNF-α, and ROS. This happens because of the reply to the pathogens and cell debris. The activated microglia tend to accumulate around the lesion and remove the dead neurons in diseases like Parkinsonism, AD and multiple sclerosis. Initially these activated microglia will favor the brain by removing the debris. Later, over-activated microglia may cause neuronal injury (Xu et al. 2016; Yang et al. 2017). RS, being a potential anti-inflammatory agent, has revealed its potency in inflammatory brain damage, by activating the M2 polarization and decreasing, M1 (Yang et al. 2017). Kodali et al. (2015), in their research from 21-month-old F344 rats, showed that RS had suppressed the glial activation considerably, promoting hippocampal neurogenesis and preventing age-related mood dysfunction and memory loss. It was reported that, without the RS administration, there was increased microglial activation in the old rat models (Huang et al. 2011).

There are reports available about the association of neuroglial cells in the etiopathogenesis of AD. However, the exact mechanism during the onset and progression of AD is not clearly understood. In this context, this research attempts to answer a few gaps in the current understanding of the pathogenesis of AD and the possibility of the use of RS in its treatment. The present study uses DPZ as the standard regime in AD-induced animal model and compares the efficacy of RS, which is used as a treating drug. DPZ is used as a positive control drug, and its action is compared when it is administered as a combination therapy with the RS. Specific parameters of neurodegeneration and microglia and astrocyte population are being studied to evaluate the role of RS and the combination of RS with DPZ in prevention and therapeutic measure in AD. Effect of combination therapy of RS and DPZ and comparative analysis of RS at its different doses are not being studied and reported. These are novel in the scientific literature, and the findings of this comparison are of novelty.

**Fig. 1:**
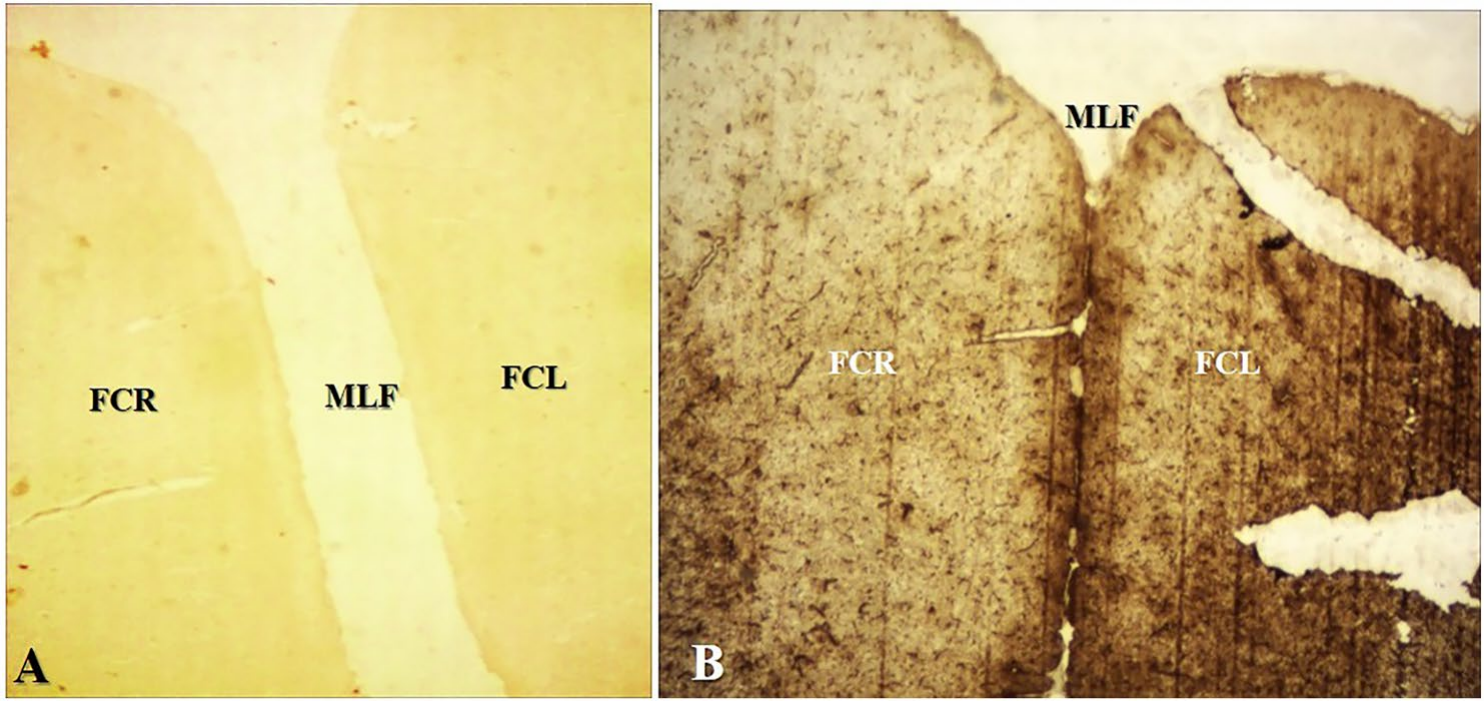
Low-power magnification (4X) view of the frontal cortex of the RS 10 treated group; **A** Immunohistochemical staining with mouse monoclonal anti-human CD 68 for microglia; **B** Immunohistochemical staining with mouse monoclonal anti-GFAP for astrocytes (*FCR* frontal cortex of right side, *FCL* frontal cortex of left side, *MLF* median longitudinal fissure of cerebral cortex)

**Fig. 2:**
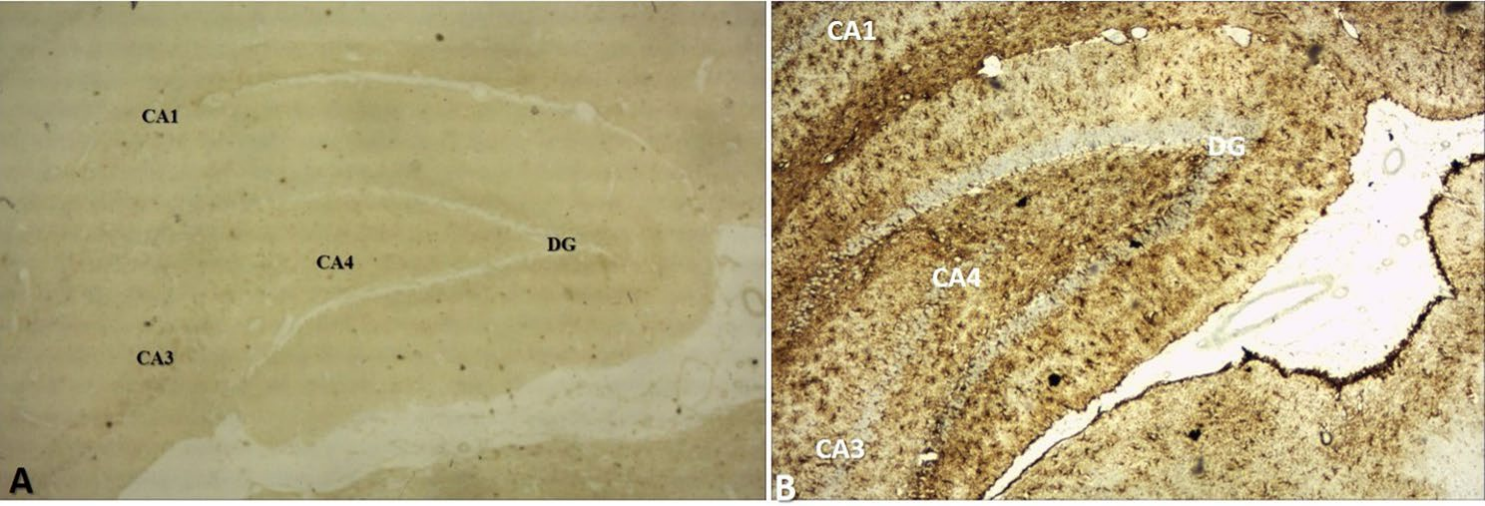
Low-power magnification (4X) view of the hippocampal regions of the RS 10 treated group; **A** Immunohistochemical staining with mouse monoclonal anti-human CD 68 for microglia; **B** Immunohistochemical staining with mouse monoclonal anti-GFAP for astrocytes (*CA1* cornu ammonis 1, *CA3* cornu ammonis 3, *CA4* cornu ammonis 4, *DG* dentate gyrus)

**Fig. 3:**
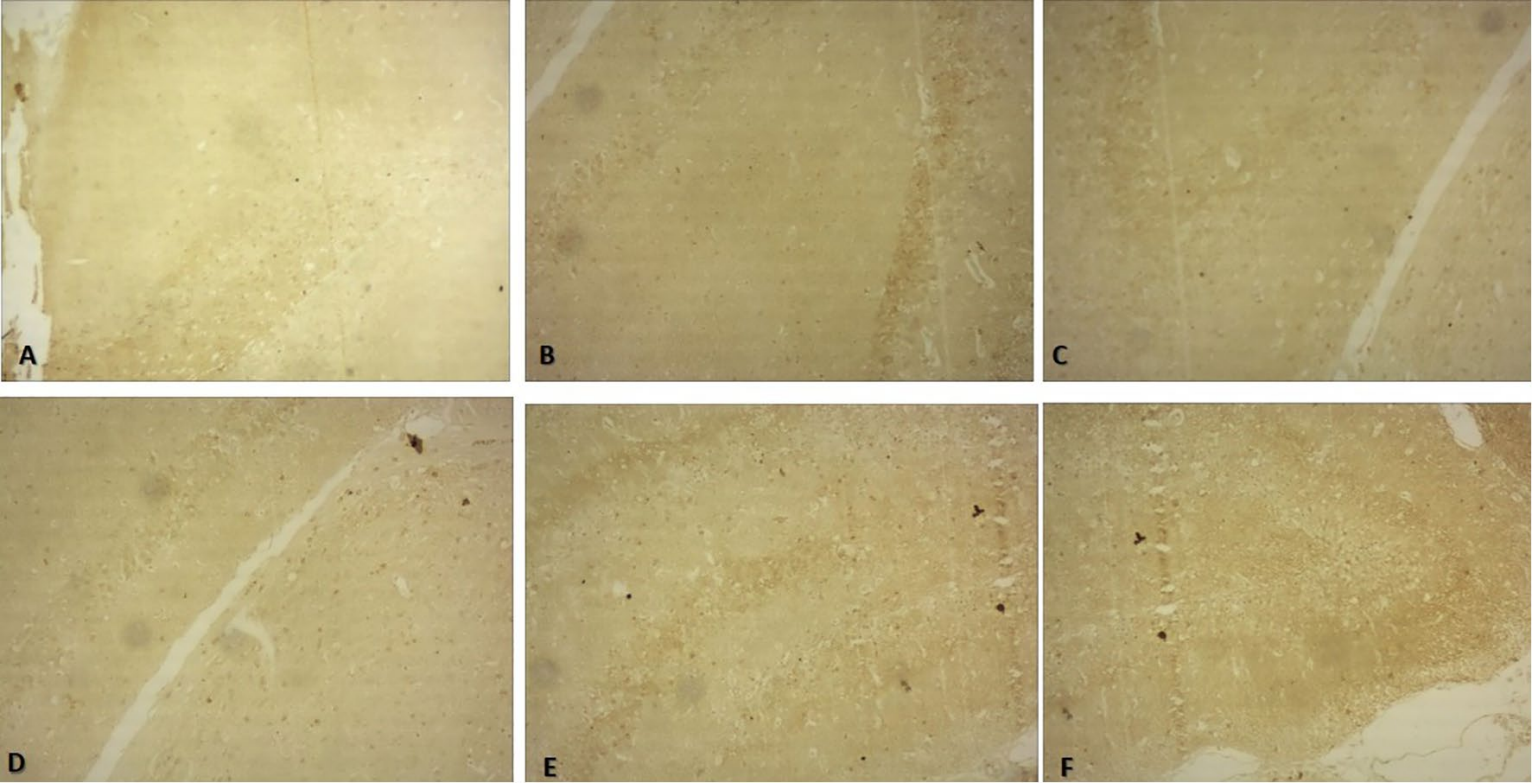
10X view of the different regions of the rat brain of the RS 10 treated group (immunohistochemical staining with mouse monoclonal anti-human CD 68 for microglia; **A **frontal cortex; **B** CA1; **C** CA2; **D** CA3; **E** CA4; **F** DG)

**Fig. 4:**
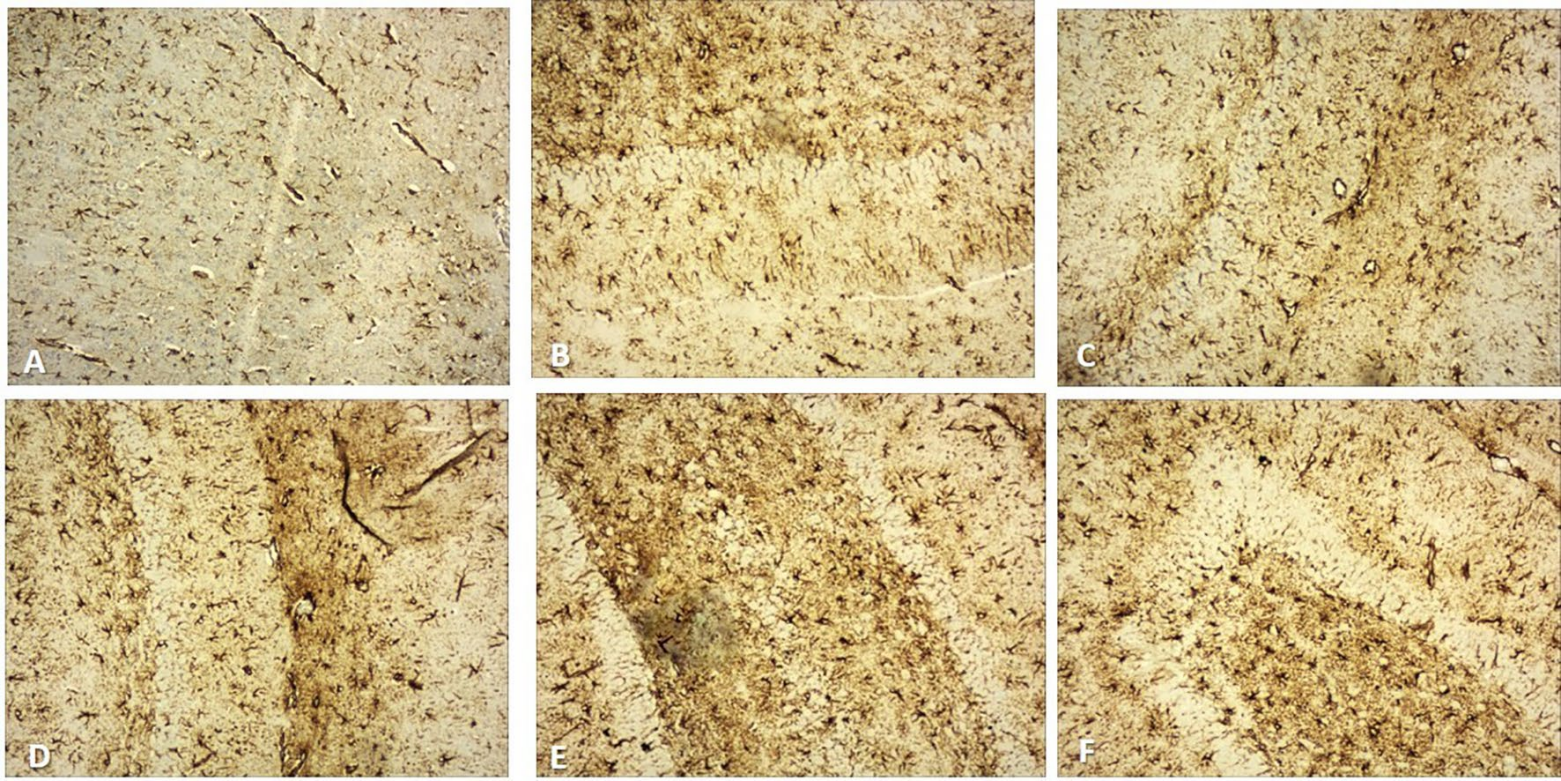
10X view of the different regions of the rat brain of the RS 10 treated group (immunohistochemical staining with mouse monoclonal anti-GFAP for astrocytes; **A** frontal cortex; **B** CA1; **C** CA2; **D** CA3; **E** CA4; **F** DG)

The principal objective of current study was to determine the effect of RS in numerical expression of microglial cells and astrocytes in the colchicine-induced AD model of *Wistar* albino rats. The secondary objective of this study was to study the same parameters at the same regions of brain by using the RS and DPZ combination therapy. The colchicine was administered intraventricularly to induce the neurodegeneration, and different doses of RS were given prophylactically and as a treatment. Frontal cortex (Fig. 1A, B), regions of hippocampus (Fig. 2A, B) were microscopically examined with the immunohistochemical staining with mouse monoclonal anti-human CD 68 (Fig. 3) and mouse monoclonal anti-GFAP (Fig. 4) for the microglia and astrocytes, respectively.

## Materials and methods

This randomized case–control study included male albino *Wistar* rats of 3 months of age, weighing between 220 and 250 gm, and each group had six rats (*n* = 6). The institutional review board approval (KMC/MNG/IAEC/22-2018) was attained before performing this research.

### Animal groups

Group 1 (AD): AD rat model (AD induced by administration of colchicine).

Group 2 (RS 10): AD model treated with RS (10 mg/kg), one-week post-surgery.

Group 3 (RS 20): AD model treated with RS (20 mg/kg), one-week post-surgery.

Group 4 (RS 10/10): AD model treated with RS (10 mg/kg), one week before and after surgery.

Group 5 (RS 20/20): AD model treated with RS (20 mg/kg), one week before and after surgery.

Group 6 (DPZ + RS): AD model treated with DPZ (1 mg/kg) and RS (10 mg /kg), one-week post-surgery.

Colchicine, RS, and DPZ were dissolved in the normal saline. RS was given through the intraperitoneal route, and DPZ was administered orally. RS was started from the first postoperative day up to 7 days in the treatment groups 2 and 3. It was given from 7 days before the surgical procedure in groups 4 and 5 and sustained until a week after the procedure. In group 6, RS and DPZ were both administered for 7 days postoperatively. Stereotaxic surgery was performed to administer the colchicine intraventricularly in the brain as per the previous publications by Madhyastha et al. (2002) and Rao et al. (2021). Decapitation was performed, and the brain was immersed in 10% formalin for two days. The embedding was done to prepare the paraffin blocks, followed by the microtomy with the rotary microtome. Sections of 6 to 7 µm thickness were taken from the posterior hippocampal region and the frontal cortex.

### Chemicals

RS (pale yellow powder, 99.9% pure form) and colchicine were received from Bengaluru, India (Sigma-Aldrich, catalog number 9754). The mouse monoclonal anti-glial fibrillary acidic protein (GFAP) and mouse monoclonal anti-human CD68 were procured from Dako, Denmark, and Produktionsverg, Denmark. Other chemicals were obtained from HPLC, Sigma (St. Louis, USA). The RS dosage was decided as per Sharma and Gupta (2002) and Wiciński et al. (2020). The DPZ dose ranges between 0.375 and 0.75 mg/kg/day as per Hernandez et al. (2006). In this study, the dosage of DPZ (1 mg/kg/day) and RS (10 mg and 20 mg/kg/day) was considered as per our previous research (Rao et al. 2021).

### Estimation of microglial cells and astrocytes

**Fig. 5:**
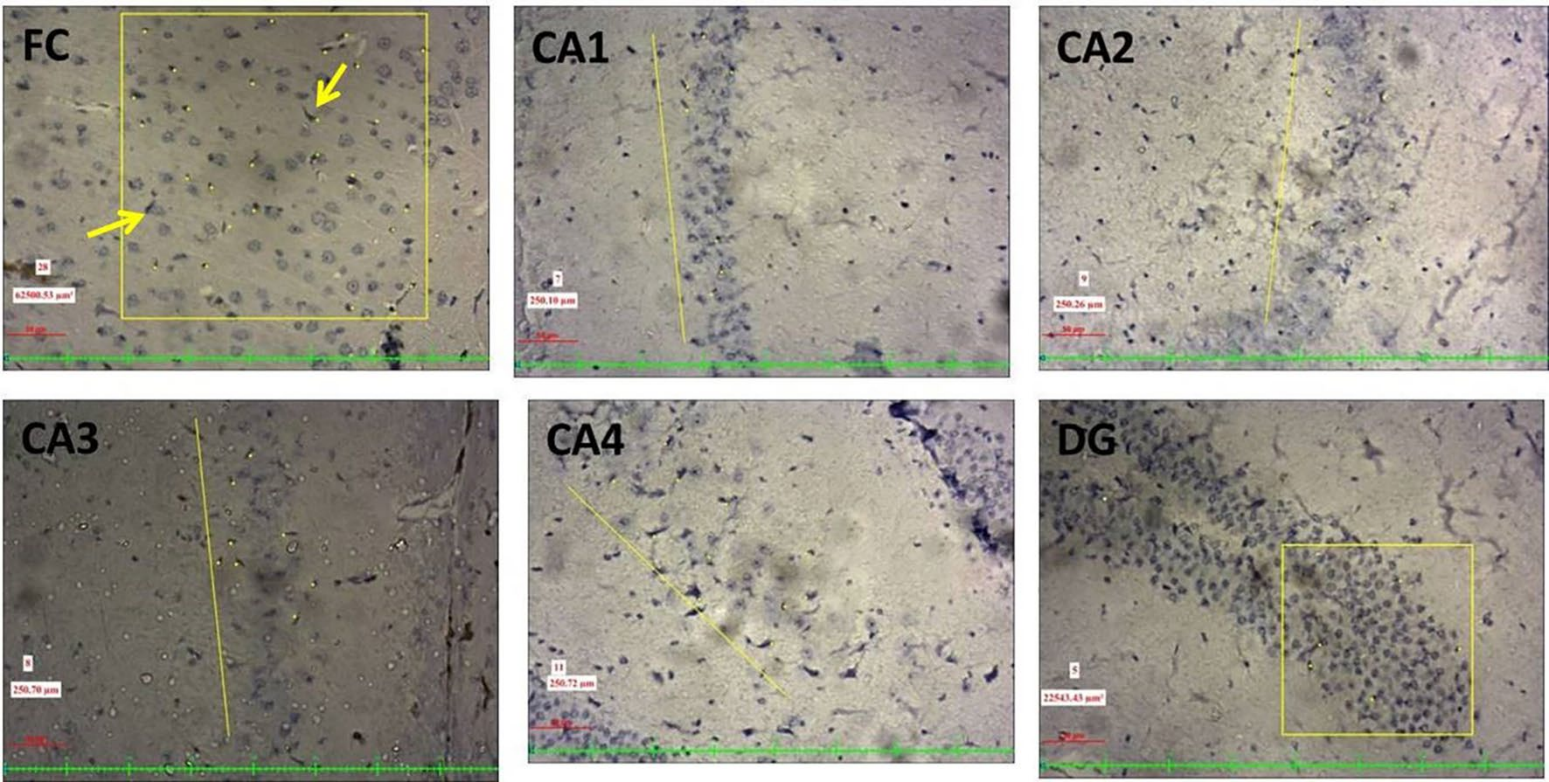
Photographs of the frontal cortex and hippocampal subregions (20X view, immunohistochemical staining with mouse monoclonal anti-human CD68; imaging software NIS Elements Br version 4.30) showing the activated microglia (marked with yellow arrows)

**Fig. 6:**
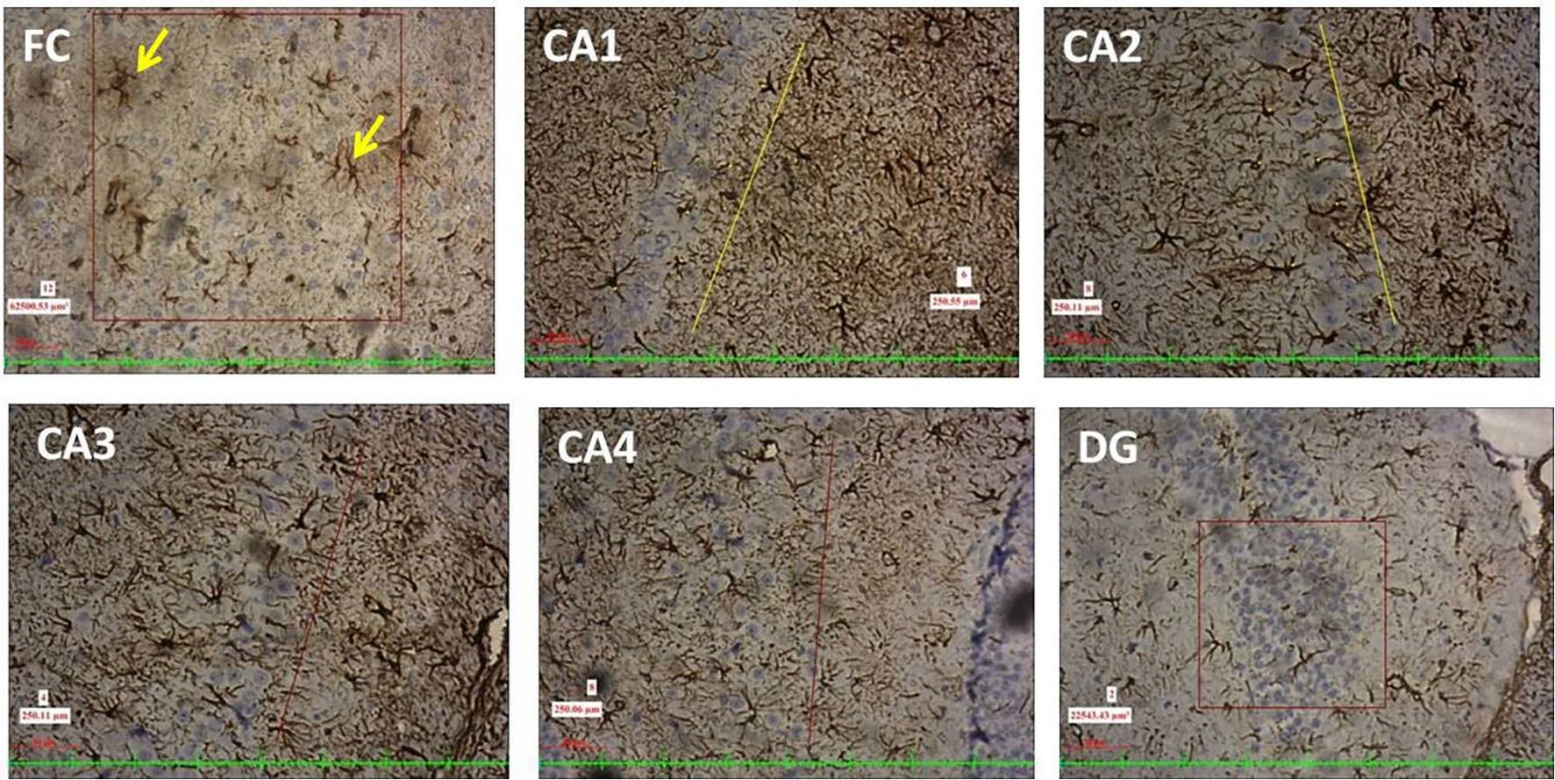
Photographs of the frontal cortex and hippocampal subregions (20X view, immunohistochemical staining with mouse monoclonal anti-GFAP; imaging software NIS Elements Br version 4.30) showing the activated astrocytes (marked with yellow arrows)

Identification and counting of microglial cells (Fig. 5) and astrocytes (Fig. 6) were done according to the protocol of Streit (1990) and Joy et al. (2019). The quantitative analysis was done under light microscopy (20X). In every section, a 250 µm^2^ area was selected for counting in the frontal cortex, whereas 250 µm length was considered in CA1, CA2, CA3 and CA4 regions and 150 µm^2^ for the dentate gyrus. The photography along with the screening was performed by using Nikon trinocular microscope (H600L), and the cells were counted by using the British version (4.30) of NIS elements imaging software as per Madhyastha et al. (2002).

### Statistical analysis

The quantitative data procured are given as mean plus or minus standard deviation. The ‘Easy R’ software (R.4.1.2 version) was utilized for performing the statistical comparison. One-way ANOVA test was applied to compare the significance of differences among the groups. The comparisons were considered highly significant if *p* < 0.001, moderately significant if *p* < 0.01, and significant if *p* < 0.05.

## Results

### Estimation of the microglia

**Fig. 7:**
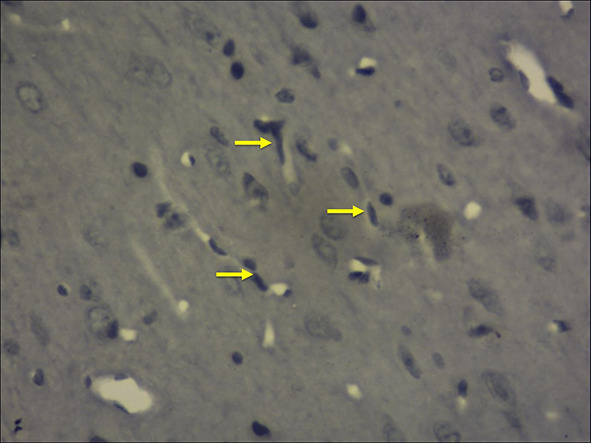
40X view of the frontal cortex with immunohistochemical stain with mouse monoclonal anti-human CD68, showing the activated microglia (marked with yellow arrows)

**Fig. 8:**
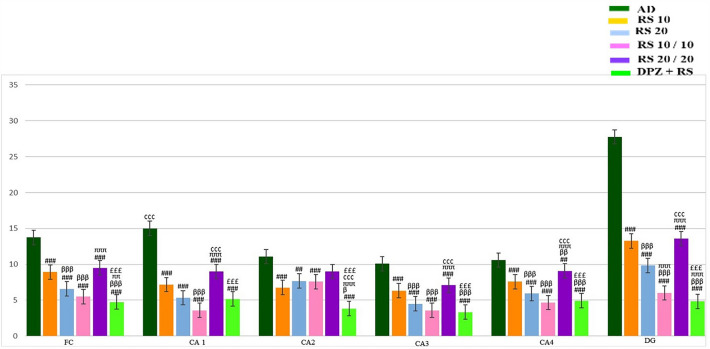
Comparison of mean values of number of microglial cells among various groups of this study in frontal cortex (FC), CA1, CA2, CA3, CA4 and dentate gyrus (DG) regions (one-way ANOVA and post hoc test, ^$^—vs control, ^$$$^—*p* < 0.001, ^$$^—*p* < 0.01, ^$^—*p* < 0.05; ^α^—vs sham OP, ^ααα^—*p* < 0.001, ^αα^—*p* < 0.01, ^α^—*p* < 0.05; ^#^—vs AD, ^###^—*p* < 0.001, ^##^—*p* < 0.01, ^#^—*p* < 0.05; ^β^—vs RS 10, ^βββ^—*p* < 0.001, ^ββ^—*p* < 0.01, ^β^—*p* < 0.05; ^π^—vs RS 20, ^πππ^—*p* < 0.001, ^ππ^—*p* < 0.01, ^π^—*p* < 0.05; ^¢^—vs RS 10/10, ^¢¢¢^—*p* < 0.001, ^¢¢^—*p* < 0.01, ^¢^—*p* < 0.05; ^£^—vs RS 20/20,^£££^—*p* < 0.001,^££^—*p* < 0.01, ^£^—*p* < 0.05; ^€^—vs DPZ, ^€€€^—*p* < 0.001, ^€€^—*p* < 0.01, ^€^—*p* < 0.05)

Figure 7 shows the high power view (40X) of the frontal cortex with immunohistochemical stain with mouse monoclonal anti-human CD68, showing the activated microglia. The activated microglia were microscopically observed at the region, away from the hippocampal and frontal cortex neurons. Mean values of microglial cells over the frontal cortex and various regions of hippocampus are symbolized in Tables 1 and 2. The comparison of mean values of microglial cells at the frontal cortex and individual areas of hippocampus is represented in Fig. 8.

**Table 1:** Mean number of microglia in frontal cortex counted in 250 μm^2^ area of various groups

Groups	Mean number of microglia in frontal cortex
AD	13.75 ± 1.35
RS 10	8.91 ± 0.99^###^
RS 20	6.58 ± 0.99^###, βββ^
RS 10/10	5.5 ± 1.08^###, βββ^
RS 20/20	9.5 ± 0.67^###, πππ^
DPZ + RS	4.75 ± 1.35^###, βββ, ππ,£££^

One-way ANOVA and post hoc test, ^#^—vs AD, ^###^—*p* < 0.001, ^##^—*p* < 0.01, ^#^—*p* < 0.05; ^β^—vs RS 10, ^βββ^—*p* < 0.001, ^ββ^—*p* < 0.01, ^β^—*p* < 0.05; ^π^—vs RS 20, ^πππ^—*p* < 0.001, ^ππ^—*p* < 0.01, ^π^—*p* < 0.05; ^¢^—vs RS 10/10, ^¢¢¢^—*p* < 0.001, ^¢¢^—*p* < 0.01, ^¢^—*p* < 0.05; ^£^—vs RS 20/20, ^£££^—*p* < 0.001, ^££^—*p* < 0.01, ^£^—*p* < 0.05

**Table 2:** Mean number of microglia counted in 250 μm length of CA1, CA2, CA3 and CA4 regions of hippocampus and 150μ^2^ of dentate gyrus

Groups	Mean number of microglia				
	CA1	CA2	CA3	CA4	DG
AD	15.0 ± 1.53	11.08 ± 1.44	10.08 ± 1.24	10.58 ± 0.99	27.75 ± 2.66
RS 10	7.16 ± 0.83^###^	6.75 ± 2.92^###^	6.33 ± 0.88^###^	7.58 ± 1.31^###^	13.25 ± 0.86^###^
RS 20	5.33 ± 2.64^###^	7.66 ± 2.64^##^	4.5 ± 1.08^###, βββ^	5.91 ± 1.5^###, βββ^	9.83 ± 0.71^###, βββ^
RS 10/10	3.58 ± 1.64^###, βββ^	7.58 ± 1.62^###,^	3.58 ± 1.08^###, βββ^	4.66 ± 1.07^###, βββ^	6.0 ± 1.34^###, βββ, πππ^
RS 20/20	9.0 ± 1.85^###, πππ, ¢¢¢^	9.0 ± 1.41	7.08 ± 0.99^###, πππ, ¢¢¢^	9.08 ± 0.99^##, ββ, πππ, ¢¢¢^	13.58 ± 2.57^###, πππ, ¢¢¢^
DPZ + RS	5.16 ± 1.02^###,£££^	3.83 ± 1.33^###, β, πππ, ¢¢¢,£££^	3.33 ± 0.88^###, βββ,£££^	4.91 ± 0.79^###, βββ,£££^	4.83 ± 0.71^###, βββ, πππ,,£££^

One-way ANOVA and post hoc test, ^#^—vs AD,^###^—*p* < 0.001,^##^—*p* < 0.01,^#^—*p* < 0.05; ^β^—vs RS 10, ^βββ^—*p* < 0.001, ^ββ^—*p* < 0.01, ^β^—*p* < 0.05; ^π^—vs RS 20, ^πππ^—*p* < 0.001, ^ππ^—*p* < 0.01, ^π^—*p* < 0.05; ^¢^—vs RS 10/10, ^¢¢¢^—*p* < 0.001, ^¢¢^—*p* < 0.01, ^¢^—*p* < 0.05; ^£^—vs RS 20/20, ^£££^—*p* < 0.001, ^££^—*p* < 0.01, ^£^—*p* < 0.05

### Estimation of microglial cells in the frontal cortex

AD group has shown increased microglia compared to all treatment groups with statistical significance (*p* < 0.001). Compared to RS 10 group, RS 20, RS10/10, DPZ + RS groups have shown significant decrease in the microglia (*p* < 0.001). Here RS 20/20 group has shown elevated microglia than RS 10 without significance (*p* > 0.05). The RS 10/10 group has shown less number of microglia, when compared to RS 20 group, without statistical significance (*p* > 0.05). DPZ + RS group also has shown less number of microglia with significance (*p* < 0.01). Compared to RS 20 group, RS 20/20 group had elevated microglia with significance (*p* < 0.001). In the DPZ + RS combination group, the microglia number was lesser than RS 10/10 group, without significance (*p* > 0.05). RS 20/20 group has shown significantly more microglia than RS 10/10 (*p* < 0.001). The DPZ + RS combination group has substantially less number of microglia than the RS 20/20 group (*p* < 0.001).

### Estimation of microglia in CA1 region

AD group has shown increased microglia compared to all treatment groups with statistical significance (*p* < 0.001). When compared to RS 10 group, RS 20, RS10/10 and DPZ + RS groups have shown decreased microglia. Here the comparison with the RS 10/10 group showed statistical significance (*p* < 0.001). RS 20/20 group had more microglia than the RS 10 group without any statistical significance (*p* > 0.05). In RS 10/10 and DPZ + RS groups, the microglia were lesser than RS 20. However, this association was statistically not significant (*p* < 0.05). RS 20/20 group had significantly more microglia than the RS 20 (*p* < 0.001). RS 20/20 and DPZ + RS groups had more microglia than the RS 10/10group. But, the comparison only with RS 10/10 group was significant (*p* < 0.001). DPZ + RS group has significantly less number of microglia than those of the RS 20/20 group (*p* < 0.001).

### Estimation of microglia in CA2 region

All the treatment groups have shown a decreased number of microglia than the group AD (*p* < 0.05), but this difference was not significant, when the statistics was applied with the RS 20/20 group (*p* > 0.05). When compared to RS 10 group, RS 20, RS10/10 and DPZ + RS groups have shown fewer microglial numbers. However, this difference was significant only with DPZ + RS (*p* < 0.05). In RS 10/10 and DPZ + RS groups, the number of microglia was less when compared to RS 20 group. Here a comparison with DPZ + RS showed statistical significance (*p* < 0.001). Though the microglial number was more in RS 20/20 than in RS 20 group, it was insignificant (*p* > 0.05). DPZ + RS combination group showed significantly less number of microglia than the RS 10/10 group (*p* < 0.001). The DPZ and RS combination group had significantly lesser number of microglia than the RS 20/20 (*p* < 0.001).

### Estimation of microglia in CA3 region

AD group has shown increased microglia compared to all treatment groups with statistical significance (*p* < 0.001). When compared to RS 10 group, RS 20, RS 10/10 group and DPZ + RS group had significantly less number of microglia (*p* < 0.001). RS 20/20 group had more microglia than the RS 10 group without any statistical significance (*p* > 0.05). In RS 10/10 group and DPZ + RS group, the number of microglia was less compared to RS 20 group. Here the comparison did not reveal a significant difference statistically (*p* > 0.05). The number of microglia was higher significantly in the RS 20/20 group than in RS 20 (*p* < 0.001). When compared to RS 10/10 group, RS 20/20 group has shown suggestively more numbers (*p* < 0.001), whereas the DPZ + RS group has shown a decrease in numbers without statistical significance (*p* > 0.05). DPZ + RS group has significantly less microglia than the RS 20/20 group (*p* < 0.001).

### Estimation of microglia in CA4 region

AD group has shown increased microglia compared to all treatment groups with statistical significance (*p* < 0.001). When compared to RS 10 group, RS 20, RS 10/10, DPZ + RS groups have shown less microglia with significance (*p* < 0.001). The RS 20/20 group has shown significantly more microglia than the RS 10 group (*p* < 0.01). In RS 10/10 group and DPZ + RS group the number of microglia was less compared to RS 20 group. Here comparison was not significant (*p* > 0.05). Microglia were significantly higher in RS 20/20 group than in RS 20 (*p* < 0.001). Compared to RS 10/10 group, RS 20/20 group and DPZ + RS groups had more number of microglia. Here the comparison was significant only with RS20/20 group (*p* < 0.001). The DPZ + RS group had less number of microglia than the RS 20/20, and this was statistically significant (*p* < 0.001).

### Estimation of microglia in the dentate gyrus

AD group has shown increased microglia compared to all treatment groups with statistical significance (*p* < 0.001). When compared to RS 10 group, RS 20, RS 10/10, DPZ + RS groups have shown less microglia with significance (*p* < 0.001). RS 20/20 group has delivered more microglia than RS 10 group without any significance (*p* > 0.05). In RS 10/10 group and DPZ + RS group the number of microglia was less compared to RS 20 group. Here comparison was statistically significant (*p* < 0.001). The microglial number was significantly more in RS 20/20 group than in RS 20 group (*p* < 0.001). In DPZ + RS group, the microglia number was lesser than RS 10/10 group, without significance (*p* > 0.05). RS 20/20 group revealed significantly more microglia than RS 10/10 (*p* < 0.001). DPZ + RS group has considerably less number of microglia than the RS 20/20 group (*p* < 0.001).

### Estimation of astrocytes

**Fig. 9:**
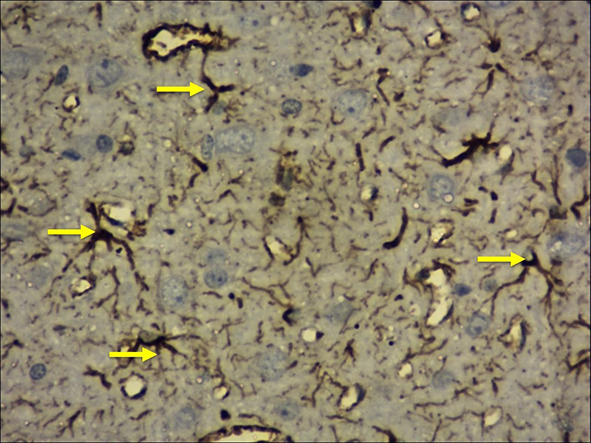
40X view of the frontal cortex with immunohistochemical stain with mouse monoclonal anti-GFAP, showing the activated astrocytes

**Fig. 10:**
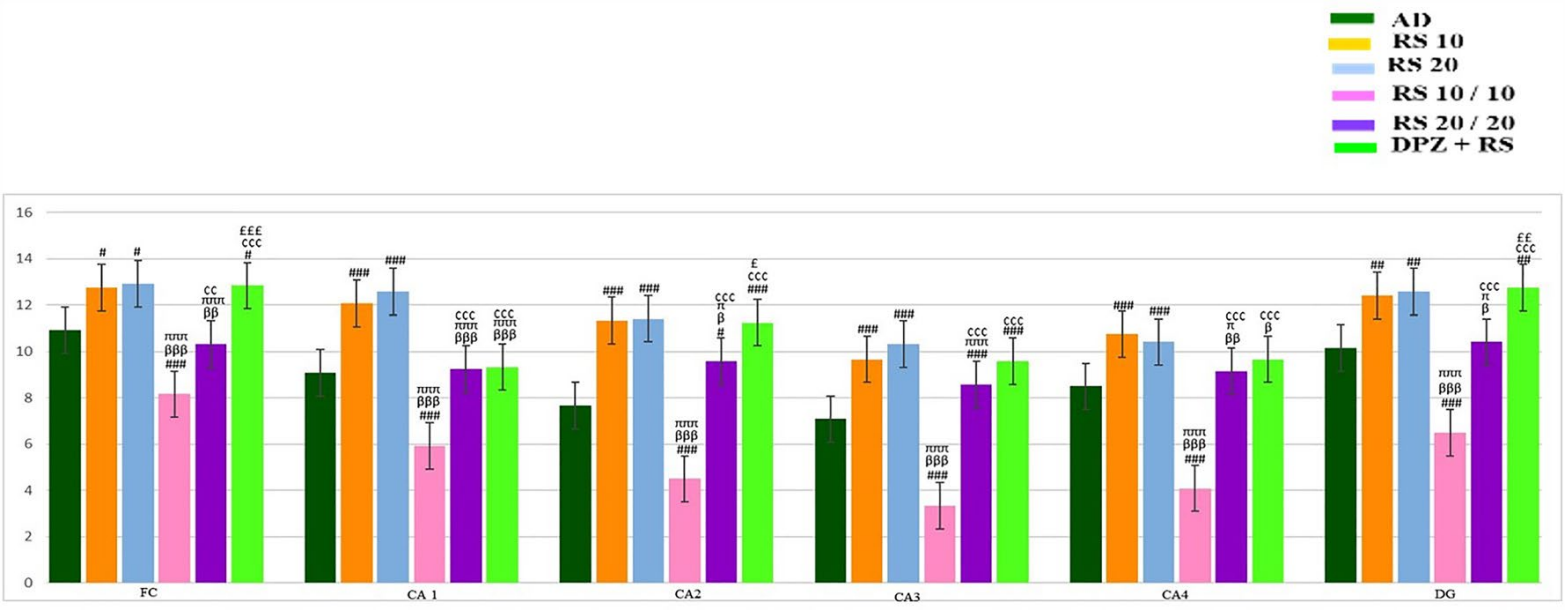
Comparison of mean values of number of astrocytes among various groups of this study in frontal cortex (FC), CA1, CA2, CA3, CA4 and dentate gyrus (DG) regions (one-way ANOVA and post hoc test, ^#^—vs AD, ^###^—*p* < 0.001, ^##^—*p* < 0.01, ^#^—*p* < 0.05; ^β^—vs RS 10, ^βββ^—*p* < 0.001, ^ββ^—*p* < 0.01, ^β^—*p* < 0.05; ^π^—vs RS 20, ^πππ^—*p* < 0.001, ^ππ^—*p* < 0.01, ^π^—*p* < 0.05; ^¢^—vs RS 10/10, ^¢¢¢^—*p* < 0.001, ^¢¢^—*p* < 0.01, ^¢^—*p* < 0.05; ^£^—vs RS 20/20, ^£££^—*p* < 0.001, ^££^—*p* < 0.01, ^£^—*p* < 0.05; ^€^—vs DPZ, ^€€€^—*p* < 0.001, ^€€^—*p* < 0.01, ^€^—*p* < 0.05)

Unlike the microglia, the astrocyte expression was more closely associated with the neurons. Figure 9 shows the high power view (40X) of the frontal cortex with immunohistochemical stain with mouse monoclonal anti-GFAP, showing the activated astrocytes. The mean values of astrocytes in the frontal region and various regions at the hippocampus are represented in Tables 3 and 4, respectively. The comparison of mean values of astrocytes at the frontal cortex and individual areas of hippocampus is represented in Fig. 10.

**Table 3:** Mean number of astrocytes in frontal cortex counted in 250 μm^2^ area of various groups

Groups	Mean number of astrocytes
AD	10.91 ± 1.50
RS 10	12.75 ± 1.71^#^
RS 20	12.91 ± 1.50^#^
RS 10 / 10	8.16 ± 1.46^###, βββ, πππ^
RS 20 / 20	10.33 ± 0.77^ββ, πππ, ¢¢^
DPZ + RS	12.83 ± 1.40^#,¢¢¢,£££^

One-way ANOVA and post hoc test, ^#^—vs AD, ^###^—*p* < 0.001,^##^—*p* < 0.01,^#^—p < 0.05;^β^—vs RS 10,^βββ^—*p* < 0.001, ^ββ^—*p* < 0.01, ^β^—*p* < 0.05; ^π^—vs RS 20, ^πππ^—*p* < 0.001, ^ππ^—*p* < 0.01, ^π^—*p* < 0.05; ^¢^—vs RS 10/10, ^¢¢¢^—*p* < 0.001, ^¢¢^—*p* < 0.01, ^¢^—*p* < 0.05; ^£^—vs RS 20/20, ^£££^—*p* < 0.001,^££^—*p* < 0.01,^£^—*p* < 0.05

**Table 4:** Mean number of astrocytes counted in 250 μm length of CA1, CA2, CA3 and CA4 regions of hippocampus and 150 μm^2^ of dentate gyrus

Groups	Mean number of astrocytes				
	CA1	CA2	CA3	CA4	DG
AD	9.08 ± 0.79	7.66 ± 1.55	7.08 ± 1.16	8.5 ± 1.0	10.16 ± 1.74
RS 10	12.08 ± 1.37^###^	11.33 ± 1.55^###^	9.66 ± 0.77^###^	10.75 ± 0.86^###^	12.41 ± 1.50^##,¢¢¢^
RS 20	12.58 ± 2.06^###^	11.41 ± 1.08^###^	10.33 ± 0.77^###^	10.41 ± 1.2^###^	12.58 ± 1.72^##,¢¢¢^
RS 10/10	5.91 ± 1.50^###, βββ, πππ^	4.5 ± 1.31^###, βββ^	3.33 ± 0.88^###, βββ, πππ^	4.08 ± 0.79^###, βββ, πππ^	6.50 ± 1.73^###, βββ, πππ^
RS 20/20	9.25 ± 0.75^βββ, πππ, ¢¢¢^	9.58 ± 1.37^#, β, π, ¢¢¢^	8.58 ± 1.08^###, πππ, ¢¢¢^	9.16 ± 1.02^ββ, π, ¢¢¢^	10.41 ± 0.79^β, π,,¢¢¢^
DPZ + RS	9.33 ± 0.77^βββ, πππ, ¢¢¢^	11.25 ± 0.96^###,¢¢¢,£^	9.58 ± 0.66^###,¢¢¢^	9.66 ± 0.65^β,¢¢¢^	12.75 ± 1.42^##,¢¢¢,££^

One-way ANOVA and post hoc test, ^#^—vs AD, ^###^—*p* < 0.001, ^##^—*p* < 0.01,^#^—*p* < 0.05;^β^—vs RS 10, ^βββ^—*p* < 0.001, ^ββ^—*p* < 0.01, ^β^—*p* < 0.05; ^π^—vs RS 20, ^πππ^—*p* < 0.001, ^ππ^—*p* < 0.01, ^π^—*p* < 0.05; ^¢^—vs RS 10/10, ^¢¢¢^—*p* < 0.001, ^¢¢^—*p* < 0.01, ^¢^—*p* < 0.05; ^£^—vs RS 20/20, ^£££^—*p* < 0.001, ^££^—*p* < 0.01, ^£^—*p* < 0.05

### Estimation of astrocytes in frontal cortex

AD group has shown decreased astrocytes than RS 10, RS20, and DPZ + RS groups with statistical significance (*p* < 0.05). RS10/10 and RS 20/20 groups have shown a decrease with respect to the number of astrocytes in comparison with the AD group. However, statistical significance was observed only with RS 10/10 group (*p* < 0.001). Compared to RS 10 group, there was increase in number, in RS20 and DPZ + RS groups. But statistically, this comparison was insignificant (*p* > 0.05). RS 10/10 (*p* < 0.001) and RS 20/20 (*p* < 0.01) groups have shown a significant increase in number than the RS 10 group. In RS 10/10, RS 20/20 and DPZ + RS groups number of astrocytes was more than in the RS 20 group. Here the statistical significance was seen only with RS10/10 and RS 20/20 groups (*p* < 0.001). RS 20/20 group and DPZ + RS groups had significantly more astrocytes than RS 10/10 group (*p* < 0.01). The DPZ + RS combination group showed more astrocytes than the RS 20/20 group, with statistical significance (*p* < 0.001).

### Estimation of astrocytes in the CA1 region

Compared to AD group, RS10, RS20, RS20/20 and DPZ + RS groups have shown significant hike in the astrocyte number. Here a comparison with RS 20/20 group was not significant statistically (*p* > 0.05). RS 10/10 group had lesser astrocytes than the AD group, which revealed significance statistically (*p* < 0.001). Compared to RS 10 group, RS 20 group revealed higher astrocytes, but it was insignificant (*p* > 0.05). RS 10/10, RS20/20 and DPZ + RS groups showed significantly more number of astrocytes than the RS 10 and RS 20 groups (*p* < 0.001). The RS 20/20 group and DPZ + RS groups had significantly more number of astrocytes than RS 10/10 group (*p* < 0.001). The DPZ and RS combination group showed more astrocytes than the RS 20/20 group, without statistical significance (*p* > 0.05).

### Estimation of astrocytes in the CA2 region

When compared to the AD group, RS10, RS 20, RS20/20 and DPZ + RS groups have shown more number of astrocytes with a higher statistically significant difference (*p* < 0.001). RS 10/10 group had number of astrocytes with statistically significant result (*p* < 0.001). RS 20 group had more astrocytes than the RS 10 group, without any significance (*p* > 0.05). RS 10/10 (*p* < 0.001) and RS 20/20 (*p* < 0.05) groups had less number of astrocytes than RS 10 group. Astrocytes were more in RS10 group than the DPZ + RS group. However, this was not showing any statistical significance (*p* > 0.05). When compared to RS 20 group, RS 10/10, RS 20/20 and DPZ + RS groups have shown a reduced number of astrocytes. However, the comparison with the DPZ + RS was statistically not significant (*p* < 0.05). When related to RS10/10 group, RS 20/20 and DPZ + RS groups had significantly elevated astrocytes (*p* < 0.001). DPZ + RS group showed more astrocytes than RS 20/20 group, with statistical significance association (*p* < 0.05).

### Estimation of astrocytes in the CA3 region

AD set had less number of astrocytes in comparison with the treatment-administered groups, with statistical significance (*p* < 0.001). When related to RS 10 group, RS 20 group had more numbers without significance (*p* > 0.05). The RS 10/10, RS 20/20 and DPZ + RS groups showed less number of astrocytes when compared to RS 10 group. Here the comparison with RS 10/10 group had clinical significance (*p* < 0.001). When compared to RS 20 group, RS 10, RS20/20 and DPZ + RS group were having less astrocytes. Here a comparison with the DPZ + RS group showed significance (*p* < 0.001). When compared to RS 10/10 group, RS 20/20 group and DPZ + RS groups were having significant more astrocytes (*p* < 0.001). DPZ + RS group showed more astrocytes than RS 20/20 group, without statistical significance (*p* > 0.05).

### Estimation of astrocytes in the CA4 region

AD group had astrocytes lesser than RS 10 and RS 20 group with statistical significance (*p* < 0.001). Astrocytes were also lesser in the AD group than RS 20/20 group and DPZ + RS group without significance (*p* > 0.05). RS 10/10 group consisted of lesser number of astrocytes than the AD group (*p* < 0.001). Astrocytes were lesser in RS 20 than in the RS 10 group. But there was no statistical significance observed (*p* > 0.05). In RS 10/10 (*p* < 0.001), RS 20/20 (*p* < 0.01), DPZ + RS (*p* < 0.05) groups also, astrocytes were lesser than in the RS 10 group. Though the number of astrocytes was lesser in RS 10/10, RS 20/20 and DPZ + RS groups than in the RS 20 group, the statistical significance was observed only with RS 10/10 (*p* < 0.001) and RS 20/20 (*p* < 0.05) groups. When equated to RS 10/10 group, RS 20/20 and DPZ + RS groups had expressively more astrocytes (*p* < 0.05). The DPZ and RS combination group showed more astrocytes than the RS 20/20 group, without statistical significance (*p* > 0.05).

### Estimation of astrocytes in the dentate gyrus

AD group had less number of astrocytes when compared to RS 10, RS 20, RS 20/20 and DPZ + RS groups. Here comparison with RS 20/20 group was not statistically significant (*p* > 0.05). RS 10/10 group exhibited lesser number of astrocytes than AD group (*p* < 0.001). When matched to RS 10 group, RS 20 and DPZ + RS groups had more astrocytes without significance (*p* > 0.05). The RS 10/10 and RS 20/20 groups had lesser astrocytes than the RS 10 (*p* < 0.001) and RS 20 groups (*p* < 0.05). The DPZ and RS combination therapy group presented increased astrocytes than the RS 20, without significance (*p* > 0.05). RS 20/20 group and DPZ + RS groups had significantly more astrocytes than RS 10/10 group (*p* < 0.001). DPZ + RS group disclosed more astrocytes than the RS 20/20 group, without statistical significance (*p* > 0.01).

## Discussion

The hippocampal microglia are more active immunologically than the rest of the parts of the brain (Grabert et al. 2016; van Olst et al. 2020; Rao et al. 2022). Jiang et al. (2020) reported that microglial activation and cytokine storm are common in stroke. Microglial activation can lead to neurodegeneration and accumulation of soluble phosphor-tau species resulting in the microglial degeneration at the hippocampal formation of AD patients (Navarro et al. 2018). Microglia engulf the Aβ with their phagocytic action and prevent AD; hence, they are neuroprotective. Microglia are converted into a disease-associated state with the help of apo lipoprotein E. However, this has to be determined by TREM2. But the hereditary factors and old age lead to scarcity of the microglial function, eventually leading to AD. Additionally, microglia go for an inflammatory level, engulfing the synapses and releasing neurotoxic cytokines. The loss of synapses and their decline are observed in the progressive forms of AD. The microglia can be a sword with two edges and function as both helping and damaging (Rao et al. 2020). This double nature makes the AD treatment by targeting microglia difficult. Microglial stimulation can be of use in the initial stage of AD and not useful in the advanced condition (Hansen et al. 2018)). Based on the activation state, the microglia are classified into pro-inflammatory (M1) and anti-inflammatory (M2) (Wolf et al. 2017; Mee-Inta et al. 2019). Zheng et al. (2023) observed the beneficial effects of RS against multiple sclerosis by suppressing the activated microglia. It was opined that activated microglia are involved in the etiopathogenesis of a progressive form of multiple sclerosis by producing pro-inflammatory cytokines (Zheng et al. 2023). This is in favor of our results, as we also observed that RS suppressed the activated microglia in the AD brain. He et al. (2022) also observed that RS suppressed the activated microglia in their spinal cord injury model of mice. They reported that RS helped the functional recovery of the spinal cord.

Ma et al. (2020) observed that RS can inhibit the miR155. Supporting this, Yang et al. (2017) reported that RS prevented the M1 microglia and activated the M2 microglia and sickness behavior in mice. They concluded that RS could be a promising drug to treat neurodegenerative disorders due to its potency in M1/M2 polarization. Balancing the M1 and M2 phenotypes by preventing the M1 microglia along with the activation of M2 microglia will be a therapeutic strategy in neuro-inflammatory disorders (Joseph and Venero 2013). Yao et al. (2015) described that RS will inhibit microglial proliferation and proves as anti-inflammatory. Adding to the above reports, the present study also demonstrated the ability of RS in reducing the activated microglia in oxidative damage condition. In the present study of colchicine-induced AD animal model, the number of microglia was estimated with the immunohistochemical staining with primary antibody, mouse monoclonal anti-human CD 68.

When the RS was administered to the AD rats, the number of microglia was decreased in RS 10 mg, RS 20 mg and RS 10/10 mg treated groups (*p* < 0.05), whereas, when RS was given prophylactically in a higher dose of 20 mg, the microglia were not decreased significantly. The observation of results revealed that when 10 mg of RS was given prophylactically, it was as equally potent as the combination therapy of RS and DPZ. However, administration of prophylactic dose of RS may not always be feasible clinically. Hence, we suggest that the combination therapy of RS and DPZ could be the best possible modality of treating the progressed AD.

In brain injuries and neurological disorders, the astrocytes get differentiated into reactive astrocytes. Astrocytes can play a dual role as they are neuroprotective and also can be neurotoxic (Onyango et al. 2021; Preman et al. 2021; Sheeler et al. 2020; Gamage et al. 2020). The activated astrocytes may interfere with normal synapses of the neurons and eventually result in cognitive dysfunction (Garwood et al. 2017). Means et al. (2020) studied the optic nerve astrocytes in Brown Norway rats and identified mechanisms underlying the degeneration of astrocytes, which may be susceptible to pharmaco-therapeutic drugs. They suggested that this mechanism can be applied in the astrocytes of the eye and central nervous system. It was opined that identifying such mechanisms is crucial to define the new targets for the effective treatment of eye and brain disorders. Singh et al. (2021) studied the Parkinsonism animal model of adult male Sprague Dawley rats. They suggested that RS can affect dopamine neurotransmission by involving the pre- and post-synaptic proteins, dopamine and vesicular monoamine transporters. It also helps in astrocyte activation, endoplasmic reticulum stress and altered cellular bioenergetics. Fan et al. (2021) reviewed the preclinical and clinical experiments to demarcate the potential role of astrocytes, nod-like and toll-like receptors in neuronal inflammation. They described that there will modification of astrocytes, which leads to gliosis in the spinal cord injury cases. As this gliosis prevents the recovery of spinal cord injury, prevention of it helps in these cases. Fan et al. (2021) suggested that RS is effective in neuro-inflammatory modulation, which are facilitated by the astrocytes, and RS can enhance the process of recovery in the spinal cord injury. Pertusa et al. (2008) and Saez et al. (2006) reported that GDNF secreted by the astrocytes improves neuronal function, resulting in improvement in the cognitive function in aged rats, whereas overexpressed NGF by the astrocytes results in neurotoxicity and hippocampal degeneration. Sheng et al. (1997) demonstrated the correlation between the activation of astrocytes with number of NFTs. It was suggested that it is required to correlate the Aβ formation, microglial activation and astrocyte activation. The present study has correlated the astrocytes and microglial expression in the brain of the AD model and RS-treated AD rats.

It was described that mitophagy and mitochondrial dysfunction had affected neurodegenerative diseases like AD. RS is a sirtuin-activating compound, which has been shown to increase mitophagy (Rai et al. 2020). RS has anti-inflammatory, antidiabetic and antioxidant properties (Timmers et al. 2012; Howitz et al. 2003). It is basically a phytoalexin compound, predominantly available in dietary sources like grapes, groundnuts, red wine and mulberry (Li et al. 2019). DPZ is an AChE inhibitor, which increases the acetylcholine concentration in the hippocampus and prevents beta-amyloid formation (Shin et al. 2018). It was reported that the combination of an AChE inhibitor and an antioxidant can be a better therapeutic strategy and may prove to be promising in planning the ligands for treating the dementia (Srivastava et al. 2019). Here, the present research supports this opinion, as the combination therapy of RS, an antioxidant and DPZ, an AChE inhibitor, gave the best positive results against the AD animal model.

The present study observed that RS, when administered after the induction of AD, the number of astrocytes got elevated in RS 10 mg and RS 20 mg treated groups (*p* < 0.05). When compared to the number of microglia in the same groups, we can assume that these elevated reactive astrocytes have helped in the prevention of microglial activation, thereby reducing the neurodegeneration, whereas, when RS was given prophylactically, in RS 10/10 group, before the onset of AD, the astrocytic number was not increased. During the comparison of microglial number in the same groups, we can hypothesize that there was no activation of reactive astrocytes since there was no increase in the microglial number. Hence by analyzing the above findings, we can opine that RS shows its neuroprotective effect by activating the reactive astrocytes whenever the microglia are increased in number. However, a higher prophylactic dose of RS 20/20 mg also showed elevated astrocytes. This finding could be because of the elevated microglia in the higher dose of prophylactic administration.

In contrast to our findings, Cheng et al. (2015) observed that 20 mg of RS did not cause a reduction in the astrocyte expression in AD rat models, but when it was given as 40 mg and 80 mg, it decreased the astrocyte expression. However, in their study, AD was induced by ovariectomy, combined with the d-galactose injection. According to this study, it is clear that increasing the concentration of RS gradually decreases the astrocyte activation. In our study also, there was no decrease in the astrocyte number when RS was given in 10 mg and 20 mg doses. However, there was a decrease in the astrocyte activation, when the RS was given prophylactically. In the present study, mouse monoclonal anti-GFAP was used as a marker for the astrocytes and it gave the best microscopic observations in the frontal cortex and hippocampal regions of both AD and treatment groups. It was described that, compared to cortical, striatal and thalamic astrocytes, the hippocampal astrocytes show higher GFAP expression (Escartin et al. 2019; Yu et al. 2020).

However, the present study has certain limitations. Colchicine was used to induce the neuronal degeneration. It is believed that using the transgenic models of the AD may give better insight of the pathogenesis and effect of RS. The RS was given through intraperitoneal route in order to increase the bioavailability of the drug. Since DPZ is already a clinically approved drug, which is administered orally, hence the oral route was preferred. However, the different routes of administration of the two different drugs are another limitation of this current study and may have the potential bias with the confounding factor. The future scope of this research can include the studying of the probiotic nature of RS, because there were no beneficial effects with the higher dose of RS in the present study. The effect of RS can also be studied in another neurodegenerative disorder like Parkinson’s disease. The study can be better interpreted by giving the two drugs, RS and DPZ through the same route, and the comparison can be made with this study. To quantify the subtypes of astrocytes, some additional markers are also used, such as cytosolic (S 100 β, glutamine synthetase, aldolase C, ALDHL1). These additional markers are helpful in studying the morphological profile of astrocytes. Recently, astroglia-specific fluorescent reporter mice and fluorescent dye intraglial injections were used to study the morphological characters (Jahn et al. 2018; Escartin et al. 2019; Yu et al. 2020). These can also be considered as the future implications of our present study.

## Conclusion

RS administration in 10 mg and 20 mg doses has reduced the number of microglia and increased the astrocyte number in the frontal cortex and different regions of the hippocampus in the colchicine-induced AD model. The elevated reactive astrocytes have decreased the microglial population. In the prophylactic RS dose of 10 mg, there were no activation and increase in the number of astrocytes since there was no elevation in the number of microglia. The combination therapy of RS and DPZ showed the best results among all the groups studied, indicating the synergistic effect of both these drugs, which can have the best impact on the treatment of AD.

## Data Availability

The data of this study are available with the corresponding author and can be shared upon personal request.
